# Increased Silicon Acquisition in Bananas Colonized by *Rhizophagus irregularis* MUCL 41833 Reduces the Incidence of *Pseudocercospora fijiensis*

**DOI:** 10.3389/fpls.2018.01977

**Published:** 2019-01-09

**Authors:** Louis-Raymond Gbongue, Ismahen Lalaymia, Adolphe Zeze, Bruno Delvaux, Stéphane Declerck

**Affiliations:** ^1^Laboratoire de Biotechnologies Végétale et Microbienne, Unité Mixte de Recherche et d’Innovation en Sciences Agronomiques et Génie Rural, Institut National Polytechnique Felix Houphouët-Boigny, Yamoussoukro, Côte d’Ivoire; ^2^Mycology, Applied Microbiology, Earth and Life Institute, Université catholique de Louvain, Louvain-la-Neuve, Belgium; ^3^Faculté des Bioingénieurs, Earth and Life Institute – Soil Science, Université catholique de Louvain, Louvain-la-Neuve, Belgium

**Keywords:** arbuscular mycorrhizal fungi, silicon, *Pseudocercospora fijiensis*, banana, black leaf streak disease, black sigatoka

## Abstract

This work aimed to test the hypothesis that the combination of arbuscular mycorrhizal fungi (AMF) and accumulation of silicon (Si) in banana plants via its uptake and transport by the fungus reduces the incidence of Black Leaf Steak Disease (BLSD) caused by *Pseudocercospora fijiensis*.

**Methods:** A pot experiment was conducted to compare BLSD symptoms on leaves of banana plants colonized or not by the AMF *Rhizophagus irregularis* MUCL 41833 and exposed or not to Si added to the growth substrate.

**Results:** A marked increase in plant growth parameters (i.e., pseudostem diameter and height, leaf surface area, shoot, root and total dry weight) as well as accumulation of Si, P, and Ca were noticed in the AMF-colonized banana plants in presence as well as in absence of Si added to the growth substrate. Similarly Si addition to the substrate increased plant growth parameters. Leave symptoms caused by the pathogen were observed in all the treatments but were reduced in presence of AMF as well as in presence of Si added to the growth substrate. The more drastic reduction was noticed in the AMF-colonized plants with Si added to the growth substrate. The Severity Index as well as Area Under Disease Progress Curve were considerably decreased both at 21 (∼48% and 48%, respectively) and 35 days (∼21% and ∼32%, respectively) after inoculation of the pathogen as compared with non-AMF-colonized plants in absence of Si added to the substrate.

**Conclusion:** Our findings revealed that AMF-colonized banana plants grown in a subs-trate supplemented with Si were less impacted by *P. fijiensis* than non-colonized plants grown without Si added to the growth substrate. The combination of AMF-colonized banana plants (during the weaning phase or *in vitro*) with the application of Si to soil seems thus a thoughtful option to mitigate the impact of BLSD in bananas, although such strategy needs first to be evaluated under field conditions to appraise its real potential.

## Introduction

Bananas are a major food and income source for more than 400 million people in tropical and subtropical regions (Mohiuddin et al., 2014). Their cultivation is threatened by many pests and diseases, among which the ascomycete *Pseudocercospora fijiensis* (M. Morelet) Deighton (formerly known as *Mycosphaerella fijiensis* M. Morelet), is the most devastating worldwide (Churchill, 2011; Hidalgo et al., 2016). This fungus is responsible for black leaf streak disease (BLSD) also named black sigatoka (BS), and affects all cultivars that belong to the triploid cultivars AAA, AAB, and ABB genome groups (Kumakech et al., 2015) which are derived from polyploidization and interspecific hybridization of *Musa acuminata* Colla (AA genome) and *Musa balbisiana* Colla (BB genome). The disease affects the leaves, reducing their photosynthetic activity and induces premature fruits ripening, causing yield losses above 50% when the disease is not controlled (Tuo et al., 2016).

Nowadays, the most effective control measures are the use of resistant cultivars and the application of fungicides (Churchill, 2011). However, repeated application of fungicides is harmful to the environment and may lead to the emergence of resistant strains, which in turn may affect the effectiveness of the fungicides and resistance factors in the newly developed hybrids (Kablan et al., 2012).

In the last few years, alternative control measures have emerged based on the application of nature-based compounds (NBCs) such as silicon (Epstein, 1999; Fauteux et al., 2005; Rodrigues and Datnoff, 2015) as well as on the use of bio-control agents (e.g., *Bacillus* sp., *Trichoderma* sp. or arbuscular mycorrhizal fungi – AMF), globally termed BCAs (Schouteden et al., 2015). The combination of both control measures is receiving a growing interest (Garg and Bhandari, 2016) but represent only a fraction of the studies conducted to date with both control measures separately.

Silicon (Si) is gradually recognized as an important element within plants (Rodrigues and Datnoff, 2015). Its accumulation in tissues of Si-accumulator plants, such as rice, cucurbits, wheat, corn, sorghum, and banana, has been shown to help plants fend off pests and diseases (Ma and Yamaji, 2006; Liang et al., 2015) via various mechanisms (see review by Fauteux et al., 2005). Silicon is absorbed by plant roots only as monosilicic acid and accumulates in foliar tissues where it acts as a mechanical barrier that prevents penetration by leaf pathogens (Fauteux et al., 2005). Soluble Si has also been reported to stimulate the defense mechanisms of plants via the elicitation of defense genes (Ma and Yamaji, 2008). Fawe et al. (1998) demonstrated that Si-treated cucumber plants had higher chitinases, peroxidases, polyphenol oxidases and flavonoid phytoalexins activities, all of which being reported to protect plants against fungal pathogens. Similarly, Si-treated rice and wheat plants infected with blast presented a higher resistance via the increased production of glycosylated phenolics and antimicrobial products such as diterpenoid phytoalexins (Bélanger et al., 2003). Interestingly, several authors have noticed that the interruption of Si absorption by plants resulted in a decreased bio-control effect, even though it was accumulated previously (Fawe et al., 2001). This suggested the necessity for a steady availability of soluble Si to promote its continuous absorption (Heine et al., 2007).

Arbuscular mycorrhizal fungi are obligate root symbionts that form associations with an approximate of 74% of angiosperms (Brundrett, 2009). These fungi provide the plants with minerals (e.g., phosphorus) in exchange for photosynthates (i.e., sugars, lipids – Keymer et al., 2017) provided by the plants, therefore stimulating plant growth. They also influence the physiology of their host plants by modulating their responses to abiotic (Plouznikoff et al., 2016) and biotic (Whipps, 2004) stresses. Several studies have reported their role in the reduction of incidence and/or severity of soil-borne as well as leaf pathogens (Jung et al., 2012). In banana, they have been shown to decrease the incidence of nematodes such as *Radopholus similis* (Koffi et al., 2013) and *Pratylenchus goodeyi* (Elsen et al., 2003) and fungal pathogens such as *Cylindrocladium spathiphylli* (Declerck et al., 2002), *Fusarium oxysporum* var cubense (Jaizme-Vega et al., 1997) and more recently *M. fijiensis* (Oye Anda et al., 2015).

Interestingly, Oye Anda et al. (2016) recently demonstrated that pre-colonized banana plants cv. Grande Naine accumulated more Si in shoot and roots than non-mycorrhizal plants. They further suggested that this increased accumulation may represent a potential novel avenue for banana resistance to pests and diseases. Indeed, Kablan et al. (2012) have reported a reduced severity and development of BLSD in banana plants receiving Si. This increasing protection in presence of Si was also observed against *F. oxysporum* f. sp. *cubense* and *C. spathiphylli* (Vermeire et al., 2011; Fortunato et al., 2012), although none of the studies considered AMF. The aim of the present study was thus to test and validate the hypothesis that the combination of root colonization by the AMF and increased accumulation of Si in banana plants via its uptake and transport by the fungus reduces the incidence of BLSD caused by *P. fijiensis*.

## Materials and Methods

### Biological Material

Micropropagated banana plants (*Musa acuminata* Colla cv. Grande Naine, clone CV902, AAA genome, Cavendish group), susceptible to BLSD, were provided by VITROPIC SA (Montpellier, France) in hermetically sealed boxes on the Murashige-Skoog (Murashige and Skoog, 1962) medium. The culture boxes were kept for 10 days in a growth chamber at 27/25°C (day/night) with a photoperiod of 12 h day^-1^, a relative humidity (RH) of 80% and under a photosynthetic photon flux (PPF) of 300 μmol m^-2^ s^-1^ before use (Anene and Declerck, 2016).

A strain of *Rhizophagus irregularis* (Blaszk., Wubet, Renker and Buscot) C. Walker and Schuessler as [‘irregulare’] MUCL 41833 was purchased from the Glomeromycota *in vitro* collection (GINCO^1^). The fungus was cultured with maize plants var. Codibag Bio (CODISEM, Realville, France) in 1 L pots filled with a sterilized (121°C for 15 min) substrate composed of volcanic lava granules (0–3 mm diameter, DCM, Belgium) for inoculum production. The pots were placed in a greenhouse (22/18°C day/night, with a photoperiod of 16 h day^-1^, a RH of 70% and PPF of 300 μmol m^-2^s^-1^).

The strain of *Pseudocercospora fijiensis* (M. Morelet) Deighton was isolated from symptomatic leaves of French plantain type in Azaguie (Côte d’Ivoire). The *P. fijiensis* population present in this area is ranked among the most aggressive known in the world (Camara, 2011). Conidia of the fungus were trapped by the technique described by Carlier et al. (2003) and subsequently incubated in Petri plates (90 mm diam.) on 3% water-agar (Sigma-Aldrich, St. Louis, MO, United States) in the dark at 22°C. Seven-days after germination, one single conidium was transferred on 39 g L^-1^ potato dextrose agar (Scharlau Chemie S.A., Barcelona, Spain) and grown for 2 weeks. The strain was then transferred onto V8 juice agar medium (Erwin and Ribeiro, 1996) in Petri plates (90 mm diam.) for another 2 weeks under continuous light (PPF of 60–65 μmol m^-2^ s^-1^) at 22°C for mycelium and spores production (Oye Anda et al., 2015). The mycelium was then scrapped from the culture medium, grinded in 10 ml of sterile water with a mortar and pestle and passed through sterile cheesecloth (one-layer pore size of approximately 150 μm) to separate the mycelium from conidia. The conidial suspension was then concentrated by centrifugation at 3,700 rpm for 10 min at 4°C. The concentration of conidia was determined using a Fuchs-Rosenthal counting chamber and further adjusted to obtain a suspension of 103 conidia ml^-1^ (Abadie et al., 2008). The final pathogen inoculum was obtained by adding 1% (w/v) of Gelatin (Merck, Darmstadt, Germany) to the fungal suspension for better adhesion of the conidia to the leaves.

### Experimental Set-Up

#### Growth Conditions and Mycorrhization of Banana Plants

Banana plants were cleaned from the culture medium with sterile deionized water. The plants (8 ± 1 cm height with three fully developed leaves) were planted in 1 L culture pots filled with sand (1–2 mm diameter; Euroquartz, Belgium) in March 2016. The sand was previously soaked in 2 M hydrochloric acid solution for 72 h for dissolving organic elements and removing bioavailable silicon by backwashing. The sand was then rinsed thoroughly with distilled water and sterilized twice (121°C for 15 min) at 24 h intervals.

Half of the plants (i.e., 30) were inoculated with 5 g of maize roots colonized by the AMF. The other half (i.e., the controls) received the same amount of maize roots without AMF. The plants were subsequently acclimatized for a period of 8 weeks in a growth chamber at 24°C, with a photoperiod of 12 h day^-1^ and a PPF of 150 μmol m^-2^ s^-1^ during the first week and thereafter at 28/24°C (day/night), a RH of 80% and a PPF of 225 μmol m^-2^ s^-1^. During the first week, the plants received deionized water and then a nutrient solution of Long Ashton (Hewitt, 1966) gradually increased from 50 to 200 ml.

After 8 weeks, six plants of each treatment were harvested and their development (pseudostem height and diameter, leaf surface area, and biomass of shoot, roots and total plant) and root colonization were evaluated. The remaining plants (i.e., 24 with and without AMF) were transferred to 5 L pots on the same substrate as above.

#### *P. fijiensis* Inoculation and Silicon Addition

After 1 week of growth in the 5 L pots, half of the AMF-colonized and non-colonized plants were inoculated with *P. fijiensis* by spraying 1 ml of the conidial suspension on the abaxial side of the third leaf starting from the top. The other half of the plants was not inoculated.

At the time of inoculation with *P. fijiensis* and during 5 weeks, the plants were supplied twice a week with 50 ml of Long Ashton supplemented or not with 75 ppm SiO_2_. Each plant was further watered with approximately 150 ml of deionized water every week. Silicon (Si) was supplied as silicic acid (H_4_SiO_4_). The Si solution was prepared by dissolving sodium metasilicate (Na_2_SiO_3_.5H_2_O) in deionized water and leaching in an acidic cation exchanger (Amberlite^®^ IR-120) to fix Na^+^ ions.

At inoculation with the pathogen, the RH was maintained at 100% during 3 days and thereafter decreased to 90% during 1 week and finally 70% until harvest. The banana plants were further maintained in a growth chamber at 28/24°C (day/night), with a photoperiod of 12 h day^-1^ and a PPF of 225 μmol m^-2^ s^-1^.

In total, eight treatments with six replicates were considered: banana plants inoculated or not with AMF (+AMF or -AMF), supplied or not with Si (+Si or -Si) and infested or not with *P. fijiensis* (+Pf or -Pf). This gave the following combinations: +AMF +Si +Pf, +AMF +Si -Pf, +AMF -Si +Pf, +AMF -Si -Pf, -AMF +Si +Pf, -AMF +Si -Pf, -AMF -Si +Pf and -AMF -Si -Pf.

### Data Assessment

#### Plant Growth Parameters

Plants were harvested after 11 weeks of culture in the 5 L pots. Pseudostem height (PH) was estimated from the top of the rhizome to the crossing point of the last two unfurled leaves. Pseudostem diameter (PD) was measured at mid-height of the PH. Length (l) and width (w) of the last unfurled leaf were measured to estimate the leaf surface area (LSA) as LSA = 0.7 lw (Rufyikiri et al., 2000). Shoots were then separated from the roots. Shoot dry weight (SDW) as well as root dry weight (RDW) were evaluated following drying at 60°C for 72 h. Total dry weight (TDW) of the plant was the addition of SDW and RDW.

#### Mineral Analysis

The concentration and total content of silicon (Si), phosphorus (P), and calcium (Ca) in banana shoots and roots were analyzed. The dried pseudostem and leaves on one side and the roots on the other side were ground and mineral analysis conducted after calcination at 450°C for 1 day and fusion in Li-metaborate (Flux LM 100, SOCACHIM-XRF Scientific, Belgium) +Li-tetraborate (Flux LT 100, SOCACHIMXRF Scientific, Belgium) at 1,000°C (Chao and Sanzolone, 1992), followed by dissolution of fusion beads in 2 M of HNO_3_ solution. Si, P, and Ca were measured by inductively coupled plasma-atomic emission spectrometry ICP-AES (ICP-AES, Thermo Scientific).

#### Root Colonization by the AMF

Eight weeks after mycorrhization (i.e., at transfer into the 5 L pots) and at the end of the experiment (after 11 weeks of growth in presence of the pathogen), roots colonization by the AMF was estimated. Roots were cleared 45 min in KOH (10%), subsequently washed several times with deionized water and bleached for 30 min in a fresh prepared H_2_O_2_ (3.5%) solution. The roots were then stained for 30 min in a solution of blue ink (Parker Quink^®^) diluted in 5% ink-vinegar solution produced with white household vinegar (5% acetic acid) (Anene and Declerck, 2016). All the procedures were done at 70°C in a water bath. Root colonization was assessed by the method of McGonigle et al. (1990). For each replicate, a minimum of 150 root intersections were observed under a compound microscope (Olympus BH2, Olympus Optical, GmbH, Germany) at 20×–40× magnifications, to estimate total root colonization (% RC) and proportion of root length colonized by spores/vesicles (%V) and arbuscules (%A).

#### Disease Development by *P. fijiensis*

The severity index (SI) of the pathogen was rated visually at days 21 and 35 in presence or absence of AMF grown in the substrate supplemented or not with Si. The SI of the disease was determined following the method described by Gauhl et al. (2000), using the formula:

SI=∑nb × 100/(N−1)T

where, *n* = number of leaves for each scale degree, *b* = degree of scale (0 = no symptom, 1 ≤ 1% of leaf surface presenting symptoms, 2 = 1 to 5% of leaf surface presenting symptoms, 3 = 6 to 15% of leaf surface presenting symptoms, 4 = 16 to 33% of leaf surface presenting symptoms, 5 = 34 to 50% of leaf surface presenting symptoms, 6 = 51 to 100% of leaf surface presenting symptoms). *N* = number of degree used in the scale and *T* = total number of leaves assessed.

The rate of disease development was assessed at day 21 and 35 by the area under disease progress curves (AUDPC) proposed by Campbell and Madden (1990):

$$\mathrm{AUDPC}=\sum_{i=1}^{n_{i}=1}\left[(Y_{i}+Y_{i+1})/2](X_{i+1}-X_{i}\right)$$

where, *Y*_*i*_ = disease severity at the ith observation, *X*_*i*_ = time at the ith observation, *n*_*i*_ = number of times and *i* = order index for the times.

### Statistical Analysis

**FIGURE 1:**
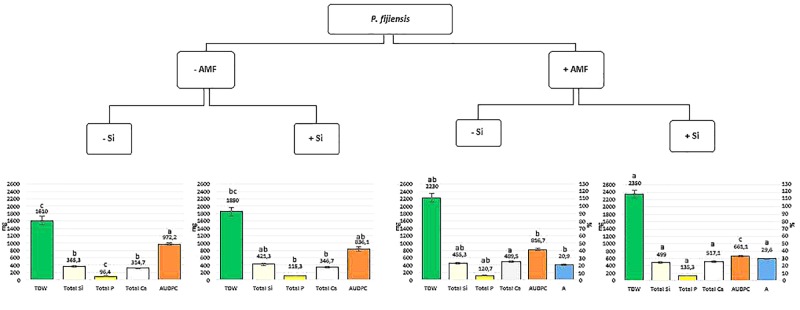
Total dry weight (TDW) (mg.10^1^), total nutrient content (mg) [silicon (Si), phosphorus (P), and calcium (Ca)] and area under disease progress curve (AUDPC) values (see vertical scale bar on the left) of banana plantlets associated (+AMF) or not (–AMF) with *Rhizophagus irregularis* MUCL 41833, and supplied or not with silicon (+Si or –Si), 35 days after their inoculation with *Pseudocercospora fijiensis* MUCL 47740. Percentage of arbuscules (A%) values (see vertical scale bar on the right) in mycorrhized banana plantlets associated supplied or not with silicon (+Si or –Si), 35 days after their inoculation with *Pseudocercospora fijiensis* MUCL 47740. For each parameter values were expressed as mean ± standard error. For each single treatment (AMF and Si) or for the combined treatments, bars with different letters are significantly different [*P* = 0.05 -global linear model-(GLM) for TDW, total Si, total P, and total Ca parameters and Kruskal–Wallis test for AUDPC parameter].

Data analyses were performed with the JMP statistical software version 14.0 from SAS (SAS Inc., Cary, NC, United States). Data for plant parameters (i.e., shoot, root and total dry weight, pseudostem diameter and height and leaf surface area), mineral (i.e., Si, P, Ca) content in shoots, roots, and total plant as well as AMF roots colonization, were analyzed using a linear general model (GLM) composed of AMF, Si and Pf factors and their interactions. To fulfill the assumptions of the method, the RDW and P root content responses were transformed using a base 10 logarithm, while the AMF root colonization (%) was normalized by arcsine transformation. Tukey’s significant difference test was used to identify the significant differences (*P* < 0.05) between treatments. Data for the SI and AUDPC were analyzed using a Kruskal–Wallis test. Dunn test, a non-parametrical test for pairwise comparison, was used to identify the significant differences (*P* ≤ 0.05) between treatments. The data presented in Figure 1 were analyzed using the GLM model for TDW, total Si, total P, and total Ca parameters and Kruskal–Wallis test for AUDPC parameter, both composed of the combination of AMF and Si factors in presence of Pf.

## Results

### Plant Growth Parameters

Throughout the experiment, the banana plants developed normally with an approximate of one new leaf emitted each week. At the end of the experiment (5 weeks after infection by the pathogen), a significant effect of the factor ‘AMF’ was noticed for all the plant growth parameters (Table 1). Indeed, whatever the addition of Si or presence of *P. fijiensis*, the PD, PH, LSA, SDW, RDW, and TDW of the plants were significantly greater in presence of AMF (Table 1). A similar effect was noticed for the factor Si. All the plant growth parameters were significantly greater in presence of Si. The factor ‘Pf’ only significantly impacted SDW (Table 1), which was significantly greater in presence of the pathogen whatever the presence of AMF or addition of Si. With the exception of the combination ‘AMF × Si’ on PD and SDW, no significant effects were noticed for any plant growth parameters for the interactions ‘AMF × Si,’ ‘AMF × Pf,’ ‘Si × Pf’ or ‘AMF × Si × Pf’ (Table 1).

**Table 1:** Growth parameters of banana plantlets inoculated or not with AMF (+AMF or -AMF), supplied or not with Si (+Si or -Si) and infested or not with *P. fijiensis* (+Pf or -Pf), estimated 5 weeks after the addition of Si and infestation by the pathogen.

Treatments	PD	PH	LSA	SDW	RDW	TDW
	(cm)	(cm)	(cm^2^)	(g)	(g)	(g)
**Single treatments**						
-AMF	2,8 ± 0,3^b^	16,1 ± 0,2^b^	209,8 ± 4,7^b^	9,3 ± 0,2^b^	7,2 ± 1^b^	16,7 ± 0,5^b^
+AMF	3,1 ± 0,3^a^	18 ± 0,2^a^	250,1 ± 4,7^a^	11,8 ± 0,2^a^	11,0 ± 1^a^	23,1 ± 0,5^a^
-Si	2,8 ± 0,3^b^	16,7 ± 0,2^b^	220,6 ± 4,7^b^	10,2 ± 0,2^b^	8,0 ± 1^b^	18,6 ± 0,5^b^
+Si	3,1 ± 0,3^a^	17,4 ± 0,2^a^	239,4 ± 4,7^a^	10,9 ± 0,2^a^	9,9 ± 1^a^	21,2 ± 0,5^a^
-Pf	2,9 ± 0,3	17,2 ± 0,2	232,8 ± 4,7	10,2 ± 0,2^b^	8,9 ± 1	19,7 ± 0,5
+Pf	2,9 ± 0,3	16,9 ± 0,2	227,2 ± 4,7	10,9 ± 0,2^a^	8,9 ± 1	20,1 ± 0,5
**Combined treatments**						
-AMF -Si -Pf	2,6 ± 0,1^b^	15,8 ± 0,3	199,9 ± 9,4	9,4 ± 0,4^de^	6,1 ± 1,1	15,6 ± 1,1
-AMF -Si +Pf	2,5 ± 0,1^b^	16 ± 0,3	193,5 ± 9,4	9,5 ± 0,4^cde^	6,5 ± 1,1	16,1 ± 1,1
-AMF +Si -Pf	3 ± 0,1^a^	16,4 ± 0,3	227 ± 9,4	8,5 ± 0,4^e^	7,8 ± 1,1	16,5 ± 1,1
-AMF +Si +Pf	3 ± 0,1^a^	16,1 ± 0,3	218,9 ± 9,4	9,8 ± 0,4^cde^	8,6 ± 1,1	18,5 ± 1,1
+AMF -Si -Pf	3,1 ± 0,1^a^	17,7 ± 0,3	246,1 ± 9,4	10,5 ± 0,4^bcd^	9,8 ± 1,1	20,6 ± 1,1
+AMF -Si +Pf	3 ± 0,1^a^	17,1 ± 0,3	242,9 ± 9,4	11,4 ± 0,4^abc^	10,6 ± 1,1	22,3 ± 1,1
+AMF +Si -Pf	3,1 ± 0,1^a^	18,9 ± 0,3	258,2 ± 9,4	12,2 ± 0,4^ab^	13,4 ± 1,1	26,2 ± 1,1
+AMF +Si +Pf	3,1 ± 0,1^a^	18,3 ± 0,3	253,4 ± 9,4	12,8 ± 0,4^a^	10,5 ± 1,1	23,5 ± 1,1

**Effect**	***p*-Value**					

AMF	**<0,0001**	**<0,0001**	**0**	**<0,0001**	**<0,0001**	**<0,0001**
Si	**<0,0001**	**0,0028**	**0,0075**	**0,0438**	**0,0042**	**0,0017**
Pf	0,3577	0,2056	0,4064	**0,0066**	0,9763	0,6188
AMF × Si	**<0,0001**	0,0705	0,2699	**0,025**	0,407	0,2263
AMF × Pf	0,8172	0,2745	0,8098	0,965	0,2316	0,2445
Si × Pf	0,0821	0,6963	0,9054	0,4475	0,2931	0,3399
AMF × Si × Pf	0,8172	0,6196	1	0,2317	0,1975	0,055


*This gave the combinations: -AMF -Si – Pf, -AMF -Si +Pf, -AMF +Si -Pf, -AMF +Si +Pf, +AMF -Si – Pf, +AMF -Si +Pf, +AMF +Si -Pf, +AMF +Si +Pf. For each growth parameter, values were expressed as mean ± standard error of six replicates. Main effects and interactions between the factors: “AMF,” “Si” and “Pf” are presented. Tests that revealed significant differences at the 0.05 α level (GLM) are indicated in bold. When two or three factors interaction was significant (*p*-value ≤ 0.05), pairwise comparisons using a Tukey’s test was performed. The results were presented in the “combined treatments” section, values followed by different letters differ significantly. When two or three factors interaction were not significant we concluded on the main effect of the single treatment (AMF, Si and Pf) effect. When the single treatment (AMF, Si and Pf) effects were significant, the values followed by two different letters in the single treatment section differ significantly. PD, pseudostem diameter; PH, pseudostem height; LSA, leaf surface area; SDW, shoot dry weight; RDW, root dry weight; TDW, total dry weight.*

### Si, P, and Ca Content of Banana Plantlets

The content of Si, P, and Ca are presented in Table 2. A significant effect of the factor ‘AMF’ was observed on Si, P and Ca content of shoots and roots (Table 2). Indeed, whatever the addition of Si or presence of *P. fijiensis*, the shoot, root and total content of Si, P, and Ca were significantly greater in the AMF-colonized banana plants. With the exception of root P and Ca contents, a significant effect was also observed for the factor ‘Si’ on Si, P, and Ca contents in plant parts with values significantly greater in the plants grown in presence of Si (Table 2). The factor ‘Pf’ only significantly impacted the shoot Ca content, with value significantly greater in presence of the pathogen (Table 2). No significant effects were noticed for Si, P, and Ca in shoots, roots or total plant for the interactions ‘AMF × Si’ or ‘Si × Pf’ (Table 2). For the interaction ‘AMF × Pf,’ a significant effect was noticed for the roots P content (Table 2). The root and total P content were significantly impacted by the interactions ‘AMF × Si × Pf’ (Table 2).

**Table 2:** Silicium (SI), phosphorus (P), and calcium (Ca) content (mg) of banana plantlets inoculated or not with AMF (+AMF or -AMF), supplied or not with Si (+Si or -Si) and infested or not with *P. fijiensis* (+Pf or -Pf), estimated 5 weeks after addition of Si and infestation by the pathogen.

Treatments	Mineral content (mg per plantlet)								
	
	Si			P			Ca		
			
	Shoot	Roots	Total Si	Shoot	Roots	Total P	Shoot	Roots	Total Ca
**Single treatments**									
-AMF	260,5 ± 9^b^	124,5 ± 6,6^b^	385 ± 11,8^b^	84,6 ± 1,8^b^	19,1 ± 1^b^	104,1 ± 2,5^b^	256,4 ± 7,9^b^	66,2 ± 3,9^b^	322,6 ± 10,2^b^
+AMF	297,9 ± 9^a^	161,5 ± 6,6^a^	459,4 ± 11,8^a^	98,1 ± 1,8^a^	29 ± 1^a^	128,4 ± 2,5^a^	400,4 ± 7,9^a^	86,3 ± 3,9^a^	486,7 ± 10,2^a^
-Si	264 ± 9^b^	128,4 ± 6,6^b^	392,5 ± 11,8^a^	85,3 ± 1,8^b^	22,4 ± 1	108,4 ± 2,5^b^	312,5 ± 7,9^b^	71,0 ± 3,9	383,5 ± 10,2^b^
+Si	294,3 ± 9^a^	152,6 ± 6,6^a^	451,9 ± 11,8^b^	97,4 ± 1,8^a^	24,7 ± 1	124,1 ± 2,5^a^	344,3 ± 7,9^a^	81,5 ± 3,9	425,9 ± 10,2^a^
-Pf	270,2 ± 9	138,9 ± 6,6	409,1 ± 11,8	90,63 ± 1,8	22,9 ± 1	115,6 ± 2,5	312,1 ± 7,9^b^	80,3 ± 3,9	392,4 ± 10,2
+Pf	288,2 ± 9	147,1 ± 6,6	435,2 ± 11,8	92,1 ± 1,8	24,1 ± 1	116,9 ± 2,5	344,8 ± 7,9^a^	72,2 ± 3,9	417 ± 10,2
**Combined treatments**									
-AMF ×-Si ×-Pf	230,6 ± 18	115,8 ± 13,4	346,4 ± 23,7	84,8 ± 3,7	18,1 ± 1,1^cd^	103 ± 5,1^cd^	239 ± 15,9	57 ± 7,9	296 ± 20,5
-AMF ×-Si × +Pf	263,1 ± 18	102,2 ± 13,4	365,3 ± 23,7	75,3 ± 3,7	20,6 ± 1,1^bcd^	96,4 ± 5,1^d^	256,4 ± 15,9	58,3 ± 7,9	314,7 ± 20,5
-AMF × +Si ×-Pf	273,1 ± 18	133,9 ± 13,4	407,0 ± 23,7	85,4 ± 3,7	16,0 ± 1,1^d^	101,7 ± 5,1^cd^	252,2 ± 15,9	80,9 ± 7,9	333,2 ± 20,5
-AMF × +Si × +Pf	275,1 ± 18	146,2 ± 13,4	421,3 ± 23,7	92,9 ± 3,7	22,2 ± 1,1^bcd^	115,3 ± 5,1^bcd^	278,1 ± 15,9	68,6 ± 7,9	346,7 ± 20,5
+AMF ×-Si ×-Pf	267,4 ± 18	135,5 ± 13,4	402,9 ± 23,7	88 ± 3,7	25,6 ± 1,1^abc^	114,1 ± 5,1^abc^	350,3 ± 15,9	83,4 ± 7,9	433,6 ± 20,5
+AMF ×-Si × +Pf	295 ± 18	160,6 ± 13,4	455,3 ± 23,7	93,1 ± 3,7	26,6 ± 1,1^abc^	120,2 ± 5,1^bc^	404,3 ± 15,9	85,2 ± 7,9	489,5 ± 20,5
+AMF × +Si ×-Pf	309,7 ± 18	170,5 ± 13,4	480,2 ± 23,7	104,3 ± 3,7	37,6 ± 1,1^a^	143,7 ± 5,1^a^	406,8 ± 15,9	99,9 ± 7,9	506,6 ± 20,5
+AMF × +Si × +Pf	319,5 ± 18	179,6 ± 13,4	499,1 ± 23,7	107,1 ± 3,7	27,8 ± 1,1^ab^	135,3 ± 5,1^ab^	440,3 ± 15,9	76,8 ± 7,9	517,1 ± 20,5

**Effect**	***p*-Value**								

AMF	**0,0055**	**0,0004**	**<0,0001**	**<0,0001**	**<0,0001**	**<0,0001**	**0**	**0,0009**	**<0,0001**
Si	**0,0222**	**0,0038**	**0,001**	**<0,0001**	0,156	**<0,0001**	**0,0072**	0,0675	**0,0057**
Pf	0,1657	0,3965	0,1278	0,5833	0,4464	0,7277	**0,0059**	0,1586	0,0975
AMF × Si	0,811	0,8398	0,9463	0,2639	0,0728	0,0653	0,2073	0,2516	0,5907
AMF × Pf	0,9548	0,3593	0,5742	0,3625	**0,0085**	0,533	0,3309	0,6561	0,5594
Si × Pf	0,3499	0,79	0,5743	0,1718	0,592	0,6801	0,7909	0,0941	0,3887
AMF × Si × Pf	0,8059	0,2792	0,6685	0,0778	**0,0448**	**0,0209**	0,5246	0,6156	0,4921


*This gave the combinations: -AMF -Si –Pf, -AMF -Si +Pf, -AMF +Si -Pf, -AMF +Si +Pf, +AMF -Si – Pf, +AMF -Si +Pf, +AMF +Si -Pf, +AMF +Si +Pf. For each growth parameter, values were expressed as mean ± standard error of six replicates. Main effects and interactions between the factors: “AMF,” “Si” and “Pf” are presented. Tests that revealed significant differences at the 0.05 α level (GLM) are indicated in bold. When three factors interaction was significant (*p*-value ≤ 0.05), pairwise comparisons using a Tukey’s test was performed. The results were presented in the “combined treatments” section, values followed by different letters differ significantly. When three factors and two factors interaction were not significant we concluded on the main effect of the single treatment (AMF, Si and Pf) effect. When the single treatment (AMF, Si and Pf) effects were significant, the values followed by two different letters in the single treatment section differ significantly.*

### Root Colonization by the AMF

Root colonization was estimated at the end of the mycorrhization phase in the 1 L culture pots (i.e., after 8 weeks) and at transfer of the banana plants to the 5 L pots (i.e., 5 weeks after addition of Si and infestation by the pathogen) (Table 3). The %TC, %A, and %V at the end of the mycorrhization phase was 67.3 ± 13.8, 25.4 ± 8.2, and 9.4 ± 1.9, respectively.

**Table 3:** Root colonization of pre-mycorrhized banana plantlets (+AMF) supplied or not with Si (+Si or -Si) and infested or not with *P. fijiensis* (+Pf or -Pf), estimated 5 weeks after addition of Si and infestation by the pathogen.

Treatments	% Spores/vesicles	% Arbuscules	% Colonization
**Single treatments**			
-Si	15 ± 0,4^b^	20,3 ± 0,7^b^	71,4 ± 0.9
+Si	17,1 ± 0,4^a^	30,5 ± 0,7^a^	72 ± 0.9
-Pf	16,16 ± 0,4	25,5 ± 0,7	71,3 ± 0.9
+Pf	15,9 ± 0.4	25,3 ± 0,7	72,1 ± 0.9
**Combined treatments**			
-Si -Pf	15,2 ± 0.6	19,7 ± 1	70,7 ± 1,2
-Si +Pf	14,8 ± 0.6	20,9 ± 1	72,1 ± 1,2
+Si -Pf	17,1 ± 0.6	31,3 ± 1	71,9 ± 1,2
+Si +Pf	17,1 ± 0.6	29,6 ± 1	72 ± 1,2

**Effect**	***p*-Value**		

Si	**0,0029**	**<0,0001**	0,6794
Pf	0,7406	0,9065	0,5461
Si × Pf	0,7017	0,1372	0,5978


*This gave the combinations: +AMF +Si +Pf, +AMF +Si -Pf, +AMF -Si +Pf, +AMF -Si -Pf. For each growth parameter, values were expressed as mean ± standard error of six replicates. Main effects and interactions between the factors: “AMF,” “Si” and “Pf” are presented. Tests that revealed significant differences at the 0.05 α level (GLM) are indicated in bold.*

At the end of the experiment, a significant effect of the factor ‘Si’ was noticed on the %V and %A, whatever the presence of the pathogen, while no significant effect was observed on the %TC (Table 3). Indeed, in presence of Si, the %V and %A were significantly greater (Table 3). No significant effects of the factor ‘Pf’ and the interaction ‘Si × Pf’ was noticed on %V, %A, and %TC (Table 3).

### Leaf Symptoms Caused by *Pseudocercospora fijiensis*

Leaf symptoms caused by *P. fijiensis* are presented in Table 4. All the banana plants inoculated with *P. fijiensis* showed symptoms of BLSD. In the AMF-colonized plants, the first symptoms appeared 25 and 22 days after inoculation of the pathogen in presence or absence of Si added to the substrate, respectively (data not shown). In the non-colonized AMF plants, the first symptoms were observed after 15 and 13 days in presence and absence of Si added to the substrate, respectively.

**Table 4:** Severity index (SI) and area under disease progress curve (AUDPC) values of banana plantlets associated (+AMF) or not (-AMF) with *Rhizophagus irregularis* MUCL 41833, and supplied or not with Si (+Si or -Si), 21 and 35 days after their inoculation with *Pseudocercospora fijiensis* MUCL 47740.

Treatments	Disease assessment			
	
	SI		AUDPC	
		
	Day 21	Day 35	Day 21	Day 35
**Kruskal–Wallis *p*-value**				
	**0,003**	**0,0148**	**0,003**	**0,0022**
**Combined treatments**				
-AMF -Si	58,3 ± 3,7^a^/B	80,6 ± 2,8^a^/A	612,5 ± 39,1^a^/B	972,2 ± 38,9^a^/A
-AMF +Si	47,2 ± 5,1^ab^/B	72,2 ± 3,5^ab^/A	495,8 ± 53,8^ab^/B	836,1 ± 55,6^ab^/A
+AMF -Si	47,2 ± 2,7^ab^/B	69,4 ± 2,8^ab^/A	495,8 ± 29,1^ab^/B	816,7 ± 30,1^b^/A
+AMF +Si	30,5 ± 2,7^b^/B	63,9 ± 2,8^b^/A	320,8 ± 29,1^b^/B	661,1 ± 24,6^c^/A


*For each parameter (i.e., SI and AUDPC), values were expressed as mean ± standard error. The interaction between the factors: “AMF” and “Si” are presented. *P*-values of the Kruskal–Wallis test are indicated in bold. For each combined treatment, values in a column followed by different lower case letters differ significantly (*p*-value ≤ 0.05). Similarly, for each combined treatment, values in the same line for a single parameter (i.e., SI or AUDPC) followed by different capital letters differ significantly (*p*-value ≤ 0.05).*

A significant effect of the different treatment was observed on the SI and AUDPC 21 and 35 days after inoculation of the pathogen (Table 4). However, only in the +AMF +Si treatment, the SI and AUDPC values were significantly lower as compared to the -AMF -Si treatment. The SI and AUDPC parameters significantly increased from day 21 to day 35, whatever the treatment.

## Discussion

Black leaf streak caused by *Pseudocercospora fijiensis* (formerly known as *Mycosphaerella fijiensis*) is the most serious foliar disease of bananas and plantains. Its control is mostly achieved through the use of fungicides or resistant cultivars, while the application of natural products or biocontrol microorganisms remains a challenge. In the recent years, it has been shown that the accumulation of Si in tissues help plants (e.g., rice, banana) fend off pests and diseases (Ma and Yamaji, 2008; Liang et al., 2015). Similarly, several studies have shown that AMF can reduce the incidence and/or severity of above as well as below-ground pathogens in numerous plants (Liu et al., 2007; Jung et al., 2012; Li et al., 2013). Very recently, Oye Anda et al. (2016) demonstrated that AMF-colonized banana plants accumulated substantially more Si in shoots and roots than non-colonized plants. It is thus tempting to speculate that the increasing uptake of Si by AMF and its subsequent accumulation in banana tissues may increase their resistance/tolerance to *P. fijiensis*.

Here, we demonstrated that AMF-colonized banana plants accumulated significantly more Si, P and Ca in shoots and roots than non-colonized plants and that the appearance of the first symptoms of BLSD was significantly delayed in the AMF-colonized plants in presence of Si added to the growth substrate, thereby reducing the incidence of the disease The SI as well as AUDPC were noticeably reduced both at 21 (∼48% and 48%, respectively) and 35 days (∼21% and ∼32%, respectively) after inoculation of the pathogen as compared with non-AMF-colonized plants in absence of Si added to the substrate.

Various mechanisms have been proposed to explain the increased resistance to pests and diseases of AMF-colonized plants. The most often reported are the (i) improved plant nutrition (especially P) and thus biomass production via for instance compensation of damage caused by pathogens, (ii) competition for colonization sites or photosynthates, (iii) changes in the root system and rhizosphere microbial populations, and (iv) activation of plant defense mechanisms (Whipps, 2004).

Increased biomass was often reported in banana plants colonized by AMF (Declerck et al., 1995; Oye Anda et al., 2015). This increase was usually attributed to the capacity of the extraradical AMF mycelium network to take up, translocate and transfer high amounts of nutrients (e.g., P) to the host plant cortical cells via arbuscules in exchange for photosynthates. In the present study, the growth parameters (i.e., PD, PH, LSA, SDW, RDW) as well as Si, P, and Ca content of AMF-colonized banana plants (in absence or presence of Si supplemented to the growth substrate) were significantly greater as compared to the non-colonized controls and could be an explanation for the increasing resistance/tolerance of plants to pests and diseases.

The role of P in increasing plant resistance has been reported in numerous studies (Phelps and Shand, 1995; Laliberté et al., 2015) and often attributed to a compensation of damages caused by pathogens (see review by Schouteden et al., 2015), although there is not always a positive correlation between increased P uptake and disease control (Schouteden et al., 2015). However, plants with a better nutrient status are probably able to tolerate higher pathogen pressures. Interestingly, Ca has been described as a key element of signaling events for the establishment and/or functioning of the mycorrhizal symbiosis (Navazio and Mariani, 2008). It acts as a secondary messenger in the phytoalexin synthesis pathway which is important in disease defense mechanism toward various pathogens (Faurie et al., 2009; Ahuja et al., 2011). In banana, high Ca content in leaves was associated to an increased resistance to BLSD (Holderness et al., 2000). Increased P and Ca content in AMF-colonized banana plants seems thus to play a role in the higher resistance of the plants to BLSD, without excluding any of the other mechanism cited above which were not explored in the present study.

The addition of Si to the growth substrate resulted in a significant increase of Si accumulation in plants colonized or not by the AMF. Indeed, in the substrate supplemented with Si, the relative increase of Si content in shoots and roots in the non-colonized plants was ∼15 and ∼13% after 35 days, respectively, as compared to the plants without Si added to the growth substrate and pathogen. In presence of the pathogen, this relative increase was ∼4% and 30% for roots and shoots, respectively. Similarly, the Si content in shoots and roots of the AMF-colonized banana plants was significantly greated as compared to the non-AMF-colonized plants in the presence as well as in the absence of Si and Pf. Indeed, in absence of the pathogen the relative increase was 11.8% and 21.5%, respectively, in presence of Si added to the growth substrate, and 13.8% and 14.5% in the absence of Si added to the growth substrate. This observation was also made in presence of the pathogen. The relative increase in Si in shoots and roots was 13.9% and 18.6%, respectively, in presence of Si added to the growth substrate and 10.8% and 36.3%, respectively, in absence of Si added to the growth substrate.

These results demonstrated the capacity of the banana plants to take up Si and pinpointed as well the significant role of AMF in the uptake and translocation of Si to the banana plants as earlier noticed by Oye Anda et al. (2016) in bananas and by Garg and Bhandari (2016), and Frew et al. (2017) in chickpea and sugarcane, respectively. Yet, the mechanisms involved in the increased accumulation of Si in banana plants colonized by AMF remain unclear. Interestingly, aquaporin transporters in AMF species are similar to those involved in Si uptake in plants (Li et al., 2013) and could thus be involved in improved silicon absorption in mycorrhizal plants. Higher photosynthetic activity induced by AMF colonization potentially increasing the Si uptake was reported by Wu and Xia (2006). Indeed, since Si uptake involves transporters similar to those responsible for water uptake (Ma and Yamaji, 2015), an increase in photosynthesis, and therefore in transpiration and water uptake, could increase Si uptake. Interestingly, an increase in the % arbuscules was observed in the plants grown in the substrate supplemented with Si. This increase may promote the transfer of Si to the plant cells as well as other elements (e.g., P), improving the plant growth.

The effects of Si on the increasing resistance of plants against different pathogens have been reported in several studies (Heine et al., 2007; Fortunato et al., 2012). Particularly in banana, Kablan et al. (2012) observed that plants supplied with Si had a higher Si concentration in the leaves and reduced symptoms of BLSD. Interestingly, the content of Si was higher in shoots than in roots as earlier reported by Henriet et al. (2008) as a probable consequence of plant transpiration. The Si deposition and accumulation in the leaves can act as a mechanical barrier that prevents penetration by the pathogen (Fauteux et al., 2005). A number of studies have also suggested that Si play an active role in plant stress signaling pathways leading to the expression of natural plant defense reactions (Fawe et al., 2001). For instance, Keeping and Reynolds (2009) suggested that Si could play similar roles to jasmonate and salicylate as modulators of induced resistance. However, the exact nature of the interaction between Si and biochemical pathways leading to resistance remains to be unraveled (Fauteux et al., 2005). Noticeably, the relative increase of Ca content in shoots and roots was ∼5% and ∼30%, respectively, in the treatments with Si added to the growth substrate as compared to the treatment without addition of Si. The accumulation of Ca in presence of Si has been observed in some monocotyledons, including banana, (Henriet et al., 2008; Mali and Aery, 2008). This increased Ca accumulation in presence of Si could be explained by the fact that Si and Ca may have the same binding sites in the plant cell wall (Inanaga and Okasaka, 1995).

The reduction in BLSD symptoms was significant in the AMF-colonized plants grown in the substrate supplemented with Si as compared to the non-colonized plants in absence of Si added to the substrate. If better plant nutrition and growth could partially compensate for disease symptoms, the increased content of Si could also play a major role. For instance, several studies have reported a higher resistance of cells to enzymatic degradation caused by fungal pathogen via a physical barrier (Datnoff et al., 2007). An increased activity of defense-related enzymes, such as polyphenoloxidase, peroxidase, and phenylalanine ammonia-lyase (PAL) was also reported in plants with increasing content of Si. This could induce the production of antimicrobial compounds in plants and regulate systemic signals, such as salicylic acid (SA), jasmonic acid (JA), and ethylene (ET), which are hormones essential for plant defense responses and developmental processes (Van Bockhaven et al., 2013). Similarly, AMF play a role in the induction of plant defense system by modulation in JA and SA dependent pathways (Li et al., 2013). Furthermore, the AMF is likely to have a role in the induction of hydrolytic enzymes (Pozo et al., 1999), enhanced levels of pathogenesis-related (PR) proteins and accumulation of phytoalexins (Harrison and Dixon, 1993). These plant hormones are key players in plant development and plant defense mechanisms.

In the present study, we demonstrated that banana plants colonized by the AMF *R. irregularis* MUCL 41833 and grown in a substrate supplemented with Si were less impacted by *P. fijiensis* than the non-colonized plants grown without Si added to the substrate. The TDW, Si, P, and Ca content were significantly greater and the AUDPC significantly lower in the +AMF+Si treatment as compared to the –AMF-Si treatment (Figure 1). Similarly, the AUDPC was significantly lower in the AMF-colonized plants with Si supplemented to the growth substrate as compared to the AMF-colonized plants without Si added to the substrate, clearly suggesting a synergism between both parameters (Figure 1). The exact mechanisms behind these observations remains unknown and need further investigations, but are possibly related to the mechanisms generally advanced for AMF and Si considered separately. Both factors may act synergistically, thus reducing the impact of BLSD. For instance, the high number of arbuscules in the AMF-colonized plants grown in the Si supplemented substrate increases the content of Si, P, and Ca and thus the total plant biomass, as compared to the non-colonized plants with or wihout Si added to the substrate (Figure 1) while at the same time the accumulation of Si in the cells induces a higher resistance of the banana plants to BLSD via the frequently reported mechanisms (Fauteux et al., 2005). Considering AMF and Si seems thus a thoughtful option to mitigate the impact of BLSD in banana cropping systems. As such, the pre-mycorrhization of banana plants (in the weaning phase or *in vitro*) and the application of Si within field may be part of an integrated pest management strategy in banana plantation. It is, however, obvious that such strategy needs first to be evaluated under field conditions to appraise its real potential.

## Author Contributions

L-RG contributed to the development of principal experiment, data collection, data analysis, data interpretation from preliminary and principal experiment, drafted the work, commentaries corrections, and finally approved and agreed with all aspects of the work. IL contributed to the interpretation of the data and drafted the work, commentaries and corrections, and finally approved and agreed with all aspects of the work. BD and AZ contributed to the development of the experiment and finally approved and agreed with all aspects of the work. SD substantially contributed to the conception and design of the experiments, interpretation of the data, drafted the corrections, and finally approved and agreed with all aspects of the work.

## Conflict of Interest Statement

The authors declare that the research was conducted in the absence of any commercial or financial relationships that could be construed as a potential conflict of interest.

## Footnotes

1 www.mycorrhiza.be/ginco-bel
